# A New Approach to Psychiatric Drug Approval in Europe

**DOI:** 10.1371/journal.pmed.1001530

**Published:** 2013-10-15

**Authors:** Corrado Barbui, Irene Bighelli

**Affiliations:** WHO Collaborating Centre for Research and Training in Mental Health and Service Evaluation, Department of Public Health and Community Medicine, Section of Psychiatry, University of Verona, Verona, Italy

## Abstract

Corrado Barbui and Irene Bighelli question the current rules governing registration of new medicines in Europe, using the example of psychiatric drugs, and argue that the concept of absolute efficacy should be replaced by the concept of added value whereby evidence from studies comparing a new product with an active comparator should guide the drug approval process.

*Please see later in the article for the Editors' Summary*

**Summary Points**

- Current rules governing the registration of new medicines in Europe are based on the concept of absolute efficacy, which implies that a difference against placebo, and not against an active comparator, makes a new investigational product eligible for registration.
- In areas where active agents are already available this policy opens the way for the approval of drugs that might have a less favourable profile than those on the market.
- We suggest that the concept of absolute efficacy is replaced by the concept of added value, which implies that evidence from studies comparing an investigational product with an active comparator guide the drug approval process

## Introduction

The European Medicines Agency (EMA) is responsible for the scientific evaluation of medicines developed by drug companies for use in European countries [1],[2]. After nearly 20 years of existence the EMA has proved useful in ensuring member states shift towards harmonisation of pharmaceutical procedures and simplification of the process by which a central authorisation becomes valid in all the states [3]. Any opinion expressed by the EMA on old or new products, relating to changes in therapeutic indications, approval, suspension, or withdrawal of a product, has to be accepted by all members of the European Union.

Since its establishment the EMA has issued recommendations, conceptual papers, and other official documents intended to guide the design of phase III clinical studies conducted for regulatory purposes (Table 1) [4]. In the present report we use the example of drugs for psychiatric disorders to highlight the main limitations of current rules governing the registration of new medicines in Europe. The case study of psychiatric disorders is particularly challenging, as several active treatments are already available on the market, rates of spontaneous remission are high, placebo and active treatment response is erratic and variable, and outcome measures are not clear-cut concepts. The European example may be relevant to inform regulatory policies in other systems of care.

**Table:** Table 1. Glossary of Regulatory Terminology

Terminology	Explanation
Investigational product	New medicine developed by a manufacturer
Phase III clinical study	Randomized study carried out by the manufacturer to assess the benefits and harms of an investigational product
Active comparator (active treatment or active control)	Medically effective treatment given as control condition in clinical studies
Placebo	Medically ineffectual treatment given as control condition in clinical studies
Three-arm study	Clinical study randomly allocating patients to one of the following three conditions: (1) investigational product; (2) active comparator; (3) placebo
Two-arm head-to-head study (comparative trial)	Clinical study randomly allocating patients to one of the following two conditions: (1) investigational product; (2) active comparator
Superiority to placebo	Clinical study designed to demonstrate that the difference in terms of primary outcome between the investigational product and placebo is statistically significant in favour of the new medicine
Superiority to an active comparator	Clinical study designed to demonstrate that the difference in terms of primary outcome between the investigational product and an active comparator is statistically significant in favour of the new medicine
Non-inferiority to an active comparator	Clinical study designed to demonstrate that the efficacy of an investigational product is not clinically inferior to an active comparator
Efficacy in absolute terms	An investigational product supported by evidence of superiority to placebo but not to an active comparator
Added value	An investigational product supported by evidence of superiority to an active comparator
Compulsory required	According to EMA guidelines, some study designs are necessary to prove the efficacy of an investigational product for a given disorder, and therefore these studies must be conducted to obtain a marketing authorization
Recommended	According to EMA guidelines, some study designs are methodologically appropriate to prove the efficacy of an investigational product for a given disorder, and therefore these studies are recommended to obtain a marketing authorization, although EMA recognises that there may be alternative designs
Acceptable	According to EMA guidelines, some study designs may be appropriate to prove the efficacy of an investigational product for a given disorder, and therefore these studies may be considered to obtain a marketing authorization, although EMA points out that there may be better alternative designs

## EMA Requirements for Drug Approval

We examined the main characteristics of the guidelines issued by the EMA on nine psychiatric disorders (Box 1). For each disorder, recommendations are issued for the acute and long-term treatment phase, and in two cases (schizophrenia and unipolar depression) requirements for treatment-resistant patients are specifically highlighted.

**Box 1. Search Strategy and Methods Used To Extract Information from European Medicine Agency Official Documents**

*Search strategy*

The EMA prepares and regularly updates scientific guidelines to help applicants prepare marketing-authorisation applications for human medicines. Guidelines provide a basis for practical harmonisation of how Member States and the Agency interpret and apply the detailed requirements for the demonstration of quality, safety, and efficacy that are in the Community directives. The Agency strongly encourages applicants and marketing-authorisation holders to follow these guidelines.

A systematic search was performed (December 2012) on the EMA's website to identify the Agency's scientific guidelines on the clinical efficacy and safety of medicines for the following psychiatric disorders: schizophrenia [26], major depression [27], bipolar disorder [28], post traumatic-stress disorder [29], insomnia [30], generalized anxiety disorder [31], panic disorder [32], social anxiety disorder [33], and obsessive compulsive disorder [34].

*Data extraction and presentation*

Working independently and in duplicate, two reviewers read the documents and extracted the following information: status of the guideline (year, draft, or adopted), phase of the psychiatric disorder (acute, long-term, treatment-resistant), primary and secondary outcomes, superiority over placebo (required or suggested or accepted by the EMA), superiority over comparator (required or suggested or accepted by the EMA), non-inferiority over comparator (required or suggested or accepted by the EMA), choice of the comparator, patient population. A tabular approach to data presentation was employed.

In terms of study design for the acute treatment phase in schizophrenia, unipolar depression, bipolar mania, bipolar depression, post-traumatic stress disorder, generalized anxiety disorder, panic disorder, social anxiety disorder, and obsessive-compulsive disorder the EMA recommends the development of three-arm studies comparing the new product with placebo and an active comparator. With such a study design, a demonstration of superiority to placebo is explicitly required, while a demonstration of non-inferiority to an active comparator is not a compulsory requirement (Table S1). As an alternative design for schizophrenia and panic disorder only, two-arm head-to-head studies to demonstrate superiority of the new drug to an active comparator are considered acceptable by the EMA. In the acute treatment of insomnia, the EMA similarly requires three-arm studies, but in this condition both a demonstration of superiority to placebo and a demonstration of non-inferiority to an active comparator is compulsorily required (Table S1).

In treatment-resistant unipolar depression the EMA requires superiority to an active comparator with a similar pharmacological profile. In treatment-resistant schizophrenia two-arm studies to demonstrate superiority to an active comparator are recommended or, alternatively, two-arm studies to demonstrate non-inferiority to clozapine.

In terms of study design for the long-term treatment phase, the EMA suggests the development of two-arm placebo-controlled withdrawal studies, where responders to an open trial of the new treatment are randomly allocated to placebo versus continuation with the same treatment, in order to demonstrate superiority to placebo. In schizophrenia and bipolar disorder, as an alternative design, non-inferiority to an active comparator is considered acceptable.

## Problems with EMA Guidelines

The present review of EMA official guidance on the design of phase III studies conducted for regulatory purposes showed that in Europe the requirement of a difference against placebo, and not against an active comparator, makes a new drug eligible for registration. Although in situations where no standard treatments are available this issue may not be relevant, in areas where active agents are available this policy may open the way to the approval of drugs that might have a less favourable profile than those already on the market. The EMA, however, points out that its mission is to approve medicines with an unambiguous demonstration of efficacy [5]. Therefore, in clinical conditions characterized by high variability in response rates a placebo arm for comparison is needed for assay sensitivity. In two-arm head-to-head comparative studies it may be impossible to differentiate similar efficacy from lack of effect of both drugs, leaving the possibility of marketing drugs without a clear and unambiguous demonstration of efficacy [5]. A second line of argument is that relying on two-arm head-to-head comparative studies leads to the approval only of drugs that are superior to those already on the market, which systematically excludes compounds that may have similar efficacy but may have some advantages in terms of overall acceptability, tolerability or adverse effects [5].

Superiority to placebo is not an a priori position of the EMA, as the Agency recognises that decisions should be on a case-by-case basis [5]. For example, in treatment-resistant schizophrenia and treatment-resistant depression superiority over an active compound is required, but this requirement is based on the assumption that antidepressants and antipsychotics are not effective in these patient populations, with the exception of clozapine. If clozapine is used as an active comparator, then a demonstration of non-inferiority makes a new antipsychotic eligible for registration in treatment-resistant schizophrenia. We note that EMA reasoning on placebo versus active comparator is not consistent. Placebo is required in conditions where several active treatments are already in use. Superiority over an active comparator is required in situations where no standard treatment is available. Non-inferiority is required in comparison with effective comparators, such as with clozapine [6].

With respect to withdrawal studies, we note that while they are believed to minimize the number of participants who are exposed to placebo, they inevitably produce results that may be applied only to patients with a response to the medication but not to those who experience spontaneous recovery. In addition, withdrawal symptoms may lead to an overestimate of the true effect of the medication. On these grounds, Deshauer and colleagues, who conducted a systematic review to examine the efficacy of long-term therapy with selective serotonin reuptake inhibitors relative to placebo in the treatment of unipolar depression, included only studies with classical two-arm parallel randomized controlled trial design, and excluded discontinuation trials [7]. They found that the efficacy of long-term treatment was much lower than that observed in previous reviews dominated by studies with discontinuation designs [7].

Another challenge is that the policy of requiring a placebo arm for comparison may decrease the external validity of study findings. Physicians may be reluctant to enter severely ill cases into a randomised process, which may result in a placebo treatment for several weeks [8]. This reluctance may similarly apply to patients with a long history of illness, who may have been exposed to several pharmacological treatments in the past, and to recent cases, where untreated symptoms may worsen functional outcomes. As a consequence, in Europe, severely ill patients may risk becoming an “orphan” population without evidence-based options.

## Time for a New Regulatory Strategy

On the basis of these challenges, we suggest the following regulatory changes that can apply to all disorders where effective agents are already in use (Table 2).

**Table 2:** Summary of the main features of suggested EMA rules that should govern the approval of new medicines for disorders where effective medicines are already in use.

Proposal	Reason	Study Design	Primary Outcome
Superiority over placebo	To provide unambiguous evidence of efficacy	Three-arm studies (new drug, placebo, active comparator)	Explanatory measure of efficacy
Non-inferiority over comparator	To ensure that new but inferior treatments do not enter the market		
Superiority over comparator	To establish the added value of the new drug on a specific outcome	At least one two-arm head-to-head study (new drug, active comparator)	Pragmatic measure of effectiveness, acceptability or safety

1. The EMA might require three-arm studies to demonstrate superiority of the new product against placebo and non-inferiority against an active comparator. Current three-arm studies submitted to the EMA cannot establish the value of the new drug in relation to the reference drug, as can be inferred from the absence of statistical power to detect a difference between active treatments [9]. In a recent EMA reflection paper the need for active control was strongly recommended for a proper evaluation of the harms and benefits of a new medicine, although this need has not been translated into policy [5]. Similarly, in the United States, the Food and Drug Administration (FDA) has recently expressed analogous views on the potential role of non-inferiority clinical trials [10]. Non-inferiority trials, by testing whether a new product is no worse than a product already in use, force manufacturers to design three-arm studies powered not only to establish superiority over placebo but also to establish non-inferiority on the basis of a pre-defined maximum difference between treatments considered as negligible. Despite some methodological weaknesses associated with this study design [11]–[14], the main benefit is that new medicines that are less effective than others already on the market would not obtain a marketing authorization.
2. In addition to three-arm trials, the EMA might require at least one two-arm head-to-head comparative trial in order to demonstrate superiority of the investigational product over an active comparator. This benefit may be documented with respect to efficacy, acceptability, tolerability, or adverse effects. Considering that current outcome measures in psychiatry have been criticised for being highly subjective, with a risk of bias connected to difficulties in maintaining double-blinding [15], we would suggest using withdrawal rates as a pragmatic outcome, since they represent a hard measure that combines the benefits and the harms of drugs [16]. The use of pragmatic primary outcomes may give a practical insight into the potential place in therapy of the new investigational product among others already in use. Consequently, choice of the active comparator might reflect the selected primary outcome, and not its pharmacological profile as the EMA suggests. Such a two-arm design, without placebo, represents a unique opportunity to enrol severely ill patients, making the study findings applicable to real-world patient populations. In the United States, although the value of including comparative efficacy and tolerability into the FDA approval process has often been highlighted [13],[17],[18], current FDA legislation does not allow an application to be refused if a new investigational product demonstrated superiority over placebo but not over an active comparator. By contrast, the World Health Organization (WHO) essential medicines list, an inventory of medicines that treat pressing global health concerns [19], or the WHO Interagency Emergency Health Kit, a list of essential medicines and medical devices urgently needed in disaster situations [20], are updated on the basis of a systematic appraisal of comparative studies.

## Implementation

The degree of implementation of these regulatory suggestions might vary according to the EMA's willingness to change [21]. European regulators, as well as regulators acting in other health care systems, should be aware that the current EMA rules have led to a market characterised by an increasing number of medicines with the same therapeutic indication [9],[22]–[24]. We argue that the long-term sustainability of such a system is a major concern, as National Health Systems (NHSs) are currently asked to sustain the financial implications of a regulatory policy that has been producing a great number of me-too drugs, which are expensive without providing additional benefit for patients and wider society. In the field of antidepressants, for example, the case of agomelatine is instructive [25]. Agomelatine was approved by the EMA on the basis of superiority to placebo, although comparative studies suggested that it may be less effective than some other antidepressants, and tolerability data highlighted clinically relevant liver toxicity. In Italy, agomelatine entered the market but, on the basis of these challenges, the Italian Medicine Agency refused to reimburse its acquisition costs, and currently patients have to pay the full price to be treated with this antidepressant.

The EMA in Europe, and regulatory authorities acting in other health care systems with similar rules, might implement a stepped strategy aiming to switch gradually from the current concept of absolute efficacy to a concept of added value. As a first step, the EMA can require three-arm studies powered to demonstrate non-inferiority, and two-arm comparative studies powered to demonstrate superiority of the new product over a reference one. This step results in new drugs that are superior to placebo but fail to show non-inferiority not being registered. New drugs that are less effective than others already on the marked are not accepted.

A second step for the EMA is to require two complementary types of phase III studies, one providing a demonstration of superiority over placebo and non-inferiority over an active comparator, and another providing a demonstration of superiority against an active comparator (Table 2). This policy meets both the needs of demonstrating efficacy unambiguously and of showing added value for a new product.

As a final step, for disorders where effective medicines are already in use, the EMA can allow only two-arm head-to-head superiority trials.

We recognise that these regulatory changes will have cost implications for drug development as well as implications in terms of length of premarketing studies, but the expected outcome is approval of medicines that only represent a real advancet in terms of harms and benefits.

## Supporting Information

Table S1 **Summary of the main requirements of the EMA on the conduct of phase III studies in psychiatric disorders.**
(DOCX)
