# Supplementary material for: A New Approach to Psychiatric Drug Approval in Europe
Source: PLoS Med. 2013 Oct 15;10(10):e1001530. doi: 10.1371/journal.pmed.1001530 (PMC3797138; doi:10.1371/journal.pmed.1001530)
Supplement: Table S1 — Summary of the main requirements of the EMA on the conduct of phase III studies in psychiatric disorders. (DOCX) [file pmed.1001530.s001.docx]

**Time to revisit the requirements for psychiatric drug approval in Europe**

Corrado Barbui and Irene Bighelli

*WHO Collaborating Centre for Research and Training in Mental Health and Service Evaluation, Department of Public Health and Community Medicine, Section of Psychiatry, University of Verona, Verona, Italy*

**Table S1**

**Table S1. Summary of the main requirements of the EMA on the conduct of phase III studies in psychiatric disorders**

|  | |  | | TRIAL DESIGN | | | |  |
| --- | --- | --- | --- | --- | --- | --- | --- | --- |
| PSYCHIATRIC DISORDER | | OUTCOMES | | PLACEBO | COMPARATOR | | |  |
| Condition (Status, year) | Treatment Phase | Primary | Secondary | Superiority | Superiority | Non-inferiority | Choice | Patient population |
| Schizophrenia  (ADOPTED, 2012) | Acute (6 weeks) | PANSS/BPRS: change in symptoms from baseline to endpoint | Responders (30% reduction at PANSS), CGI, other rating scales, dropouts, adverse events | ●●● | ● | ●● | Proven efficacy, similar pharmacological profile | At least 20% with a history of less than 5 years |
|  | Long-term (6 months) | Relapse rate | Dropouts, adverse events | ●●● |  | ●● | Same as in acute phase | Same as in acute phase |
|  | Resistant patients | Same as in acute phase | Same as in acute phase |  | ●●● | ●● | A drug with which treatment failure was documented (superiority); clozapine (non-inferiority) | Lack of improvement with at least two antipsychotics, including one atypical |
| Unipolar depression  (DRAFT, 2012) | Acute (6 weeks) | HDRS/MADRS: change in symptoms from baseline to endpoint | Responders (30% reduction at rating scale), CGI, other rating scales, dropouts, adverse events | ●●● |  | ●● | Standard antidepressant | Moderately ill patients, mainly outpatients |
|  | Long-term (6 months) | Relapse rate | Dropouts, adverse events | ●●● |  |  | Not mentioned | Same as in acute phase |
|  | Resistant patients | Same as in acute phase | Same as in acute phase |  | ●●● |  | Standard antidepressant (which is expected to have no effect) | Lack of improvement despite the use of adequate doses of at least two antidepressants |
| Bipolar disorder (ADOPTED, 2001) | Manic episode (3-4 weeks) | YMRS/BRMAS: change in symptoms from baseline to endpoint (although responders are also mentioned, unclear) | Global impression scale, responders, remitters, dropouts, adverse events | ●●● |  | ●● | Standard active treatment | Patients should be off medication with anti manic properties |
|  | Prevention (12 months) | Proportion of patients developing mania or depression | Dropouts, adverse events | ●●● |  | ●● | Standard active treatment (lithium) | Patients free of episodes for a sustained period of time |
|  | Bipolar depression (6-8 weeks) | Same as in unipolar depression | Same as in unipolar depression | ●●● |  | ●● | Standard active treatment | Patients with major depression as part of a bipolar disorder |
| PTSD (ADOPTED, 2009) | Acute (10-12 weeks) | CAPS: change in symptoms from baseline to endpoint | Responders, remitters, CGI, other rating scales, dropouts, adverse events | ●●● |  | ●● | Standard active treatment |  |
|  | Long-term (unclear duration) | Relapse rate | Dropouts, adverse events | ●●● |  |  | Not mentioned | Same as in acute phase |
| Insomnia (ADOPTED, 2011) | Acute (2-4 weeks) | Subjective sleep parameters (self-rating scales) | Sleep laboratory parameters (polysomnography), dropouts, adverse events | ●●● |  | ●●● | Standard active treatment | Patients with primary insomnia only |
|  | Long-term (6 months) | Relapse rate | Dropouts, adverse events | ●●● |  |  | Not mentioned | Same as in acute phase |
| GAD (ADOPTED, 2005) | Acute (8 weeks) | HAM-A: change in symptoms from baseline to endpoint | Responders, remitters, CGI, other rating scales, dropouts, adverse events | ●●● |  | ●● | Standard active treatment approved for GAD | Patients with GAD only, without comorbidities |
|  | Long-term (2-6 months) | Relapse rate | Dropouts, adverse events | ●●● |  |  | Not mentioned | Same as in acute phase |
| Panic Disorder (ADOPTED, 2005) | Acute (8 weeks) | PDSS/PAS: change in symptoms from baseline to endpoint | frequency and severity of panic attacks, responders, remitters, CGI, other rating scales, dropouts, adverse events | ●●● | ● | ●● | Standard active treatment approved for Panic disorder | Patients with Panic disorder only, without comorbidities |
|  | Long-term (6 months) | Relapse rate | Dropouts, adverse events | ●●● |  |  | Not mentioned | Same as in acute phase |
| Social anxiety disorder (ADOPTED, 2006) | Acute (12 weeks) | LSAS/BSPS: change in symptoms from baseline to endpoint | CGI, other rating scales, dropouts, adverse events | ●●● |  | ●● | Standard active treatment approved for Social anxiety disorder | Patients with Social anxiety disorder only, without comorbidities |
|  | Long-term (2-6 months) | Relapse rate | Dropouts, adverse events | ●●● |  |  | Not mentioned | Same as in acute phase |
| Obsessive-compulsive disorder (ADOPTED, 2005) | Acute (10-14 weeks) | Y-BOCS: change in symptoms from baseline to endpoint | Responders, remitters, CGI, other rating scales, dropouts, adverse events | ●●● |  | ●● | Standard active treatment approved for Obsessive-compulsive disorder | Patients with Obsessive-compulsive disorder only, without comorbidities |
|  | Long-term | Relapse rate | Dropouts, adverse events | ●●● |  |  | Not mentioned | Same as in acute phase |

*Legend*: ●●● required; ●● suggested; ● accepted

*Abbreviations*: PTSD: Post-Traumatic Stress Disorder; GAD: Generalized Anxiety Disorder; PANSS: Positive and Negative Syndrome Scale; BPRS: Brief Psychiatric Rating Scale; CGI: Clinical Global Impression; HDRS: Hamilton Depression Rating Scale; MADRS: Montgomery-Asberg Depression Scale; YMRS: Young Mania Rating Scale; BRMAS: Bech-Rafaelsen Mania Rating Scale; CAPS: Clinician-Administered PTSD Scale; HAM-A: Hamilton Anxiety Rating Scale; PDSS: Panic Disorder Severity Scale; PAS: Panic Attack Scale; LSAS: Liebowitz Social Anxiety Scale; BSPS; Brief Social Phobia Scale; Y-BOCS: Yale-Brown Obsessive-Compulsive Scale
